# Diagnostic Yield of Endoscopic Ultrasound-Guided Liver Biopsy in Comparison to Percutaneous Liver Biopsy: A Meta-Analysis of Randomized Controlled Trials and Trial Sequential Analysis

**DOI:** 10.3390/diagnostics14121238

**Published:** 2024-06-12

**Authors:** Megha Bhandari, Jayanta Samanta, Marco Spadaccini, Alessandro Fugazza, Stefano Francesco Crinò, Paraskevas Gkolfakis, Konstantinos Triantafyllou, Jahnvi Dhar, Marcello Maida, Nicola Pugliese, Cesare Hassan, Alessandro Repici, Alessio Aghemo, Gaetano Serviddio, Antonio Facciorusso

**Affiliations:** 1 Hepatology Department, Cambridge University Hospital, Cambridge CB4 1GN, UK; mb2023@cam.ac.uk; 2 Gastroenterology, Post Graduate Institute of Medical Education and Research, Chandigarh 160012, India; dj_samanta@yahoo.co.in (J.S.); jahnvi3012@gmail.com (J.D.); 3 Endoscopy Unit, Department of Biomedical Sciences, Humanitas University, Humanitas Clinical and Research Center IRCCS, Rozzano, 20089 Milan, Italy; marco.spadaccini@humanitas.it (M.S.); alessandro.fugazza@humanitas.it (A.F.); nicola.pugliese@humanitas.it (N.P.); cesare.hassan@hunimed.eu (C.H.); alessandro.repici@hunimed.eu (A.R.); alessio.aghemo@hunimed.eu (A.A.); 4 Diagnostic and Interventional Endoscopy of Pancreas, Pancreas Institute, University of Verona, 37134 Verona, Italy; stefanocrino@hotmail.com; 5 Department of Gastroenterology, General Hospital of Nea Ionia “Konstantopoulio-Patision”, 142 33 Athens, Greece; pgolfakis@gmail.com; 6 Hepatogastroenterology Unit, Second Department of Internal Medicine-Propaedeutic, Medical School, National and Kapodistrian University of Athens, “Attikon” University General Hospital, 124 62 Athens, Greece; ktriant@med.uoa.gr; 7 Faculty of Medicine, “Kore” University of Enna, 94100 Enna, Italy; marcello.maida@hotmail.it; 8 Gastroenterology Unit, Department of Medical and Surgical Sciences, University of Foggia, Via L Pinto 1, 71122 Foggia, Italy; gaetano.serviddio@unifg.it

**Keywords:** EUS, FNB, FNA, adequacy, tissue, cirrhosis

## Abstract

Background: The efficacy of endoscopic ultrasound-guided liver biopsy (EUS-LB) compared to percutaneous liver biopsy (PC-LB) remains uncertain. Methods: Our data consist of randomized controlled trials (RCTs) comparing EUS-LB to PC-LB, found through a literature search via PubMed/Medline and Embase. The primary outcome was sample adequacy, whereas secondary outcomes were longest and total lengths of tissue specimens, diagnostic accuracy, and number of complete portal tracts (CPTs). Results: Sample adequacy did not significantly differ between EUS-LB and PC-LB (risk ratio [RR] 1.18; 95% confidence interval [CI] 0.58–2.38; *p* = 0.65), with very low evidence quality and inadequate sample size as per trial sequential analysis (TSA). The two techniques were equivalent with respect to diagnostic accuracy (RR: 1; CI: 0.95–1.05; *p* = 0.88), mean number of complete portal tracts (mean difference: 2.29, −4.08 to 8.66; *p* = 0.48), and total specimen length (mean difference: −0.51, −20.92 to 19.9; *p* = 0.96). The mean maximum specimen length was significantly longer in the PC-LB group (mean difference: −3.11, −5.51 to −0.71; *p* = 0.01), and TSA showed that the required information size was reached. Conclusion: EUS-LB and PC-LB are comparable in terms of diagnostic performance although PC-LB provides longer non-fragmented specimens.

## 1. Introduction

Although non-invasive liver stiffness measurement has gained increasing importance in the diagnostic algorithms of several hepatic parenchymal disorders, procurement of tissue samples through liver biopsy (LB) still plays a pivotal role, particularly in the case of focal lesions. Moreover, specific liver disorders such as autoimmune hepatitis or cases with increased liver enzymes of unclear etiology require histology for a definitive diagnosis [1,2]. Historically, the typical approach for LB was represented by the percutaneous route (PC-LB), under computed tomography (CT) scan or ultrasonographic (US) guidance, with the transjugular (TJ-LB) approach representing an alternative in the presence of a contraindication to PC-LB or for difficult locations [3,4].

Endoscopic ultrasound-guided (EUS) tissue acquisition through fine-needle aspiration (FNA) or fine-needle biopsy (FNB) plays a pivotal in the diagnostic algorithms of pancreatic and abdominal solid lesions [5,6,7,8]. In recent years, an interest was raised towards EUS-guided liver biopsy either for focal lesions or for parenchymal disease, as shown in a recent meta-analysis finding a histologic diagnostic rate of 93.9% and low complication rate of 2.3% [9]. These interesting data were further improved with end-cutting FNB needles, such as the Franseen needle (Acquire^®^ [Boston Scientific, Marlborough, MA, USA]) and the fork-tip needle (SharkCore^®^ [Medtronic, Dublin, Ireland]), as reported in a recent series [10].

However, previous reports showed conflicting data on the comparison between EUS-LB and PC-LB in patients with parenchymal liver disease. Indeed, while a preliminary small randomized controlled trial (RCT) and another multicenter retrospective series suggested a higher quality for the samples with PC-LB [11,12], more recent RCTs showed spectacular results completely in favor of EUS-LB, both in terms of procurement of optimal biopsy cores and of specimen length [13,14]. Although two previous meta-analyses concluded the non-superiority of one technique over the other [15,16], these findings should be interpreted with caution based on the pooled analysis of both RCTs and retrospective studies and a limited number of included patients. According to the GRADE methodology, only a meta-analysis of RCTs can provide high-quality evidence supporting the comparison between two interventions; hence, given the recent publication of several RCTs in this field [13,14,17], we decided to perform a meta-analysis of only RCTs to better inform forthcoming clinical guidelines. Furthermore, given the low number of RCTs comparing the two techniques, a trial sequential analysis (TSA) was performed to assess the credibility of our findings. Exactly like the calculation of the sample size in individual RCTs, a TSA derives a power calculation for a meta-analysis. In TSA, studies, rather than patients, are included in chronologic order and managed as subsequent interim analyses relative to the required number of participants. This methodology allows the application of monitoring (benefit, harm, and futility) and conventional boundaries, and finally, allows the calculation of the required number of participants based on the predefined intervention effect, adjusting it for the heterogeneity observed in the included studies [18].

## 2. Materials and Methods

### 2.1. Selection Criteria

The studies included in this meta-analysis were randomized controlled trials (RCTs) meeting the following inclusion criteria: (a) patients: adult patients with liver parenchymal disorders; (b) intervention: EUS-guided liver biopsy; (c) comparator: US- or CT-guided percutaneous liver biopsy; and (d) outcomes: primary outcome was sample adequacy, whereas secondary outcomes were longest and total lengths of tissue specimens, diagnostic accuracy, and number of complete portal tracts (CPTs). We excluded (a) retrospective comparative studies, (b) single-arm studies, (c) case series, and (d) studies not reporting any of the above reported outcomes.

### 2.2. Search Strategy

The main databases such as PubMed/Medline and Embase were searched with no language restriction through December 2023, independently by two authors (AF, MB) using the following key words: (((endoscopic ultrasound [MeSH Terms]) OR (EUS [MeSH Terms])) AND (liver biopsy [MeSH Terms])). A further search was completed on additional databases (Google Scholar, Cochrane library) and browsing the references of all the review articles in the field to identify eventual further papers. When overlapping series were identified from the same study group, only the most recent and largest papers were included. The corresponding authors of the studies were reached out to to obtain missing information. The quality of the included studies was assessed by two authors independently (AF, MB) according to the Cochrane collaboration risk of bias 2 tool [19]. Any disagreements were addressed by re-evaluation and following a third opinion (CH).

### 2.3. Outcomes

The primary outcome was sample adequacy, defined according to the AASLD and British guidelines as specimen length ≥ 20 mm and number of CPTs ≥ 11 [1,20]. Diagnostic accuracy was defined as the proportion of patients correctly diagnosed using LB, hence as true positive + true negative/total number of patients. Total and maximum/longest specimen length were measured in mm and compared between EUS-LB and PC-LB along with the number of CPTs, where a CPT was defined as the presence of all 3 portal structures (portal vein, hepatic artery, and bile duct) in the sample. Safety data were analyzed only descriptively and adverse events (AEs) were graded according to the ASGE lexicon [21].

### 2.4. Statistical Analysis

Study outcomes were pooled and compared between the two groups through a random effects model based on the DerSimonian and Laird test [22], and the results were expressed in terms of risk ratio (RR) or mean difference and 95% confidence interval (CI), when appropriate. The presence of heterogeneity was calculated through I² tests with I² < 30% interpreted as low-level heterogeneity and I^2^ between 30% and 60% as moderate heterogeneity [23]. Any potential publication bias was verified through visual assessment of funnel plots. Similar to RCTs, the TSA of the meta-analyses is based on an anticipated a priori intervention effect, on the basis of which the sample size is estimated to be subsequently detected with adequate power. This sample size in the TSA is the required information size (RIS), which is the number of events (or patients) from the included studies necessary to accept or reject the a priori statistical hypothesis, adjusted for the heterogeneity among the included RCTs [18,24]. The heterogeneity-adjustment factor is calculated as the total variance in a random effects model divided by the total variance in a fixed effects model. Finally, the RIS adjusted for heterogeneity between trials (random) is calculated by multiplying the non-adjusted RIS (fixed) for the heterogeneity-adjustment factor [18]. When calculating the RIS, the type I error was set at 5% and the power at 80% whereas the anticipated incidence rates were based on the cumulative meta-analysis of RCTs. In TSA, the Z-value is updated with each additional published study added to the meta-analysis, providing the cumulative Z-curve. This Z-curve is then checked for crossover in 3 boundaries: the naïve (horizontal) boundaries, which correspond to a nominal *p* = 0.05 (|Z| 1.96); the monitoring boundaries, which are sequential monitoring boundaries calculated on the a priori intervention effect and are distinguished in benefit/harm boundaries; and the futility boundaries, which are the adjusted threshold for non-superiority and non-inferiority tests obtained by applying the α-spending function based on the O’Brien–Fleming method [18,24]. When the Z-curve crosses the naive boundaries but does not cross the monitoring boundaries, there is a statistical difference in the conventional meta-analysis but not in the TSA, thus not avoiding the risk of a false positive result. If the Z-curve lies within futility boundaries and the appropriate RIS is reached, it can be easily concluded that the intervention does not have an effect. When the result of the meta-analysis is negative and the appropriate RIS is not reached, then there is lack of power. If the Z-curve crosses monitoring boundaries, it means that the treatment has evident benefit (or harm) in respect to the control group. All statistical analyses were conducted using RevMan version 5 from the Cochrane collaboration; the TSA was conducted with the Trial Sequential Analysis 0.9.5.10 software provided by the Copenhagen Trial Unit. For all calculations, a two-tailed *p* value of less than 0.05 was considered statistically significant.

### 2.5. Quality of Evidence Assessment

The quality of evidence was assessed through the GRADE criteria [25]. Briefly, evidence from RCTs started at high quality, and was rated down for presence of any of the following factors: risk of bias in the literature, inconsistency, indirectness, imprecision, and publication bias. For imprecision, evidence was rated down even if the 95% CI crossed unity or if the optimal information size (measure of fragility) was not reached [25].

## 3. Results

### 3.1. Included Studies

**Figure 1:**
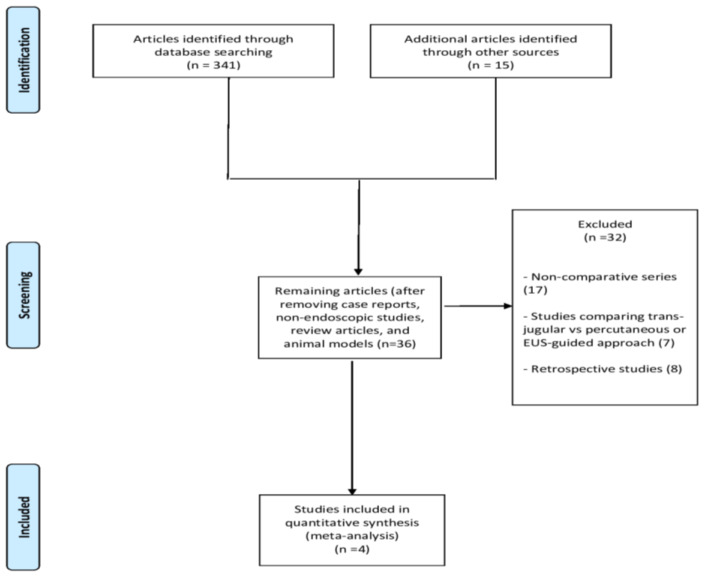
Study selection flowchart.

From 356 unique studies identified using the search strategy, we included four RCTs [11,13,14,17] (Figure 1) recruiting 258 patients, of which 129 who underwent EUS-LB and 129 PC-LB.

The main baseline characteristics of the included studies are summarized in Table 1.

Two RCTs were conducted in the USA [11,17], one in Spain [13], and one in India [14]. The recruitment period ranged from 2019 to 2022. All studies were published as full text papers except the study by Samanta et al. that was published as a conference abstract [14]; however, the authors of the current meta-analysis had full access to the dataset of this study. Baseline patient- and lesion-related characteristics were well balanced between the two study groups, with females representing the majority of participants in the included studies, while mean age ranged from 37.04 to 60.8 years. Parenchymal liver disease, including abnormal liver function tests, represented the indication to liver biopsy in all the included studies. Number of EUS needle passes ranged from 1 to 3 and the needle used was a 19 G end-cutting FNB (Acquire^®^, Boston Scientific, Marlborough, MA, USA; or SharkCore^®,^, Medtronic, Minneapolis, MS, USA) in three studies [11,13,17], whereas the study by Samanta et al. used 19 G FNA needles [14]. On the other hand, one or two passes with 16 G or 18 G needles were performed for PC-LB, which was mainly US-guided. The definition of sample adequacy was specimen length ≥ 25 mm and number of CPTs ≥ 11 in the RCT by Bang et al. [11], specimen length ≥ 20 mm and number of CPTs ≥ 11 in two RCTs [13,14], and just number of CPTs ≥ 11 in the RCT by Ali et al. [17]. Quality assessment of the included articles is reported in Supplementary Table S1. Overall, the studies were deemed at low risk of bias.

### 3.2. Sample Adequacy

**Figure 2:**
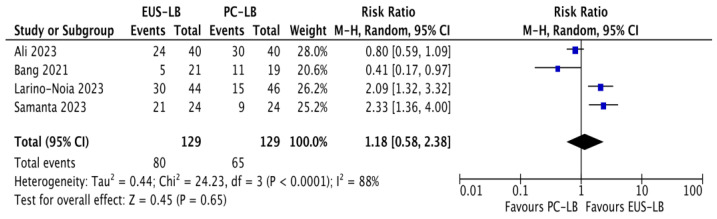
Forest plot comparing EUS-guided versus percutaneous liver biopsy in terms of sample adequacy [11,13,14,17].

Based on four RCTs [11,13,14,17], the pooled adequacy rate was 60% (35–86%) in the EUS-LB group and 51% (31–71%) in the PC-LB group, with no difference between the two approaches (RR: 1.18, CI: 0.58–2.38, *p* = 0.65; Figure 2). High heterogeneity was observed in this analysis (I^2^ = 88%).

A sensitivity analysis is reported in Supplementary Table S2. The source of heterogeneity was identified in the uneven definition of sample adequacy across the included studies. Of note, EUS-LB resulted as significantly superior when the definition as per current guidelines was used, although this finding should be interpreted with caution due to the very limited number of studies in this subgroup. No difference between EUS-LB with end-cutting needles and PC-LB was observed (RR: 0.93, CI: 0.41–2.11). No evidence of publication bias was observed through visual inspection of the funnel plot (Supplementary Figure S1a).

As reported in Supplementary Table S3, quality of evidence was rated as very low due to inconsistency (high heterogeneity), indirectness (heterogeneous definition of the outcome), and imprecision (wide confidence intervals crossing unity and failure to reach the optimal information size).

### 3.3. Other Outcomes

Table 2 reports the results of the meta-analysis of the other outcomes.

**Figure 3:**
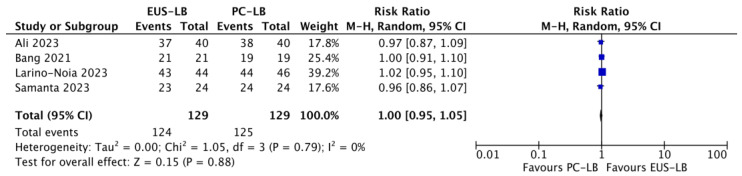
Forest plot comparing EUS-guided versus percutaneous liver biopsy in terms of diagnostic accuracy [11,13,14,17].

Based on four studies [11,13,14,17], no difference in terms of diagnostic accuracy was observed (RR: 1, CI: 0.95–1.05; *p* = 0.88) with no evidence of heterogeneity (I^2^ = 0%; Figure 3). As reported in Supplementary Table S3, moderate quality of evidence supported this analysis because of high imprecision in the estimates (wide confidence intervals crossing unity).

No difference concerning the comparison of the mean number of CPTs was registered (mean difference: 2.29, −4.08 to 8.66; *p* = 0.48), with evidence of high heterogeneity (I^2^ = 90%; Supplementary Figure S2).

Based on four studies [11,13,14,17], the mean maximum specimen length was significantly longer in the PC-LB group (mean difference −3.11, −5.51 to −0.71; *p* = 0.01), with evidence of high heterogeneity (I2 = 75%; Supplementary Figure S3). As reported in Supplementary Table S3, a moderate quality of evidence supported this analysis because of high inconsistency in the estimates (high heterogeneity).

No difference in terms of total specimen length were observed (mean difference: −0.51, −20.92 to 19.9; *p* = 0.96), with evidence of high heterogeneity (I^2^ = 95%; Supplementary Figure S4).

No evidence of publication bias was observed in any of the aforementioned analyses (Supplementary Figure S1b–e).

No major adverse events were observed in the included studies. The RCT by Larino-Noia et al. [13] reported 4 mild AEs in the EUS-LB group and 2 mild events in the PC-LB; the RCT by Samanta et al. [14] reported 5 mild events after EUS-LB and 21 mild events after PC-LB. AEs were mainly cases of abdominal pain not requiring hospitalization or specific interventions.

### 3.4. Trial Sequential Analysis

**Figure 4:**
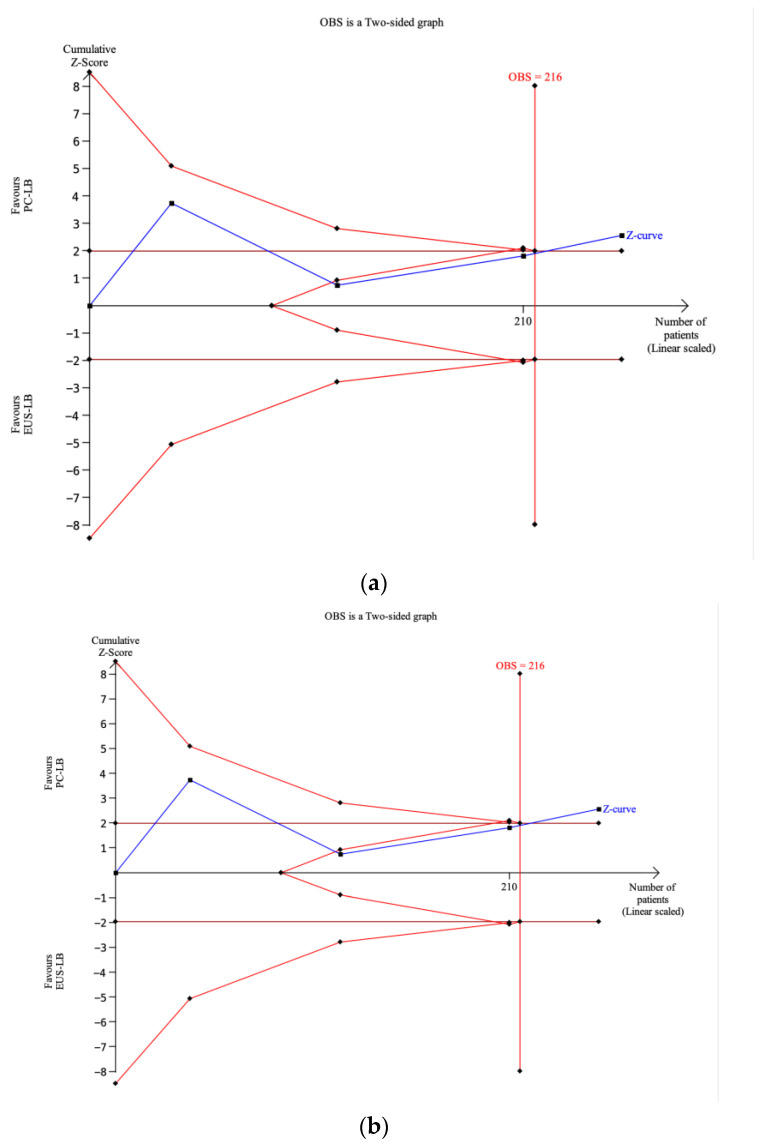
(**a**) Trial sequential analysis concerning sample adequacy. The required information size (RIS) calculated by trial sequential analysis was 1064 participants, whereas only 258 patients were actually enrolled in the included trials. The Z-curve did not cross the conventional test boundary nor the benefit monitoring boundary and remained far from the futility boundary. Therefore, there was not a statistical difference but further information is required because a false negative cannot be excluded. (**b**) Trial sequential analysis concerning max specimen length. The RIS (216 patients) was reached. The cumulative Z-curve was beyond the futility boundaries and crossed both the conventional test and the benefit monitoring boundaries, thus supporting the superiority of PC-LB over EUS-LB for this specific outcome.

As depicted in Figure 4a, the required information size (RIS) calculated by TSA for sample adequacy was 1064 participants; thus, far above the accrued information size of 258 patients enrolled in the included RCTs. The Z-curve did not cross the conventional test boundary, remaining below the benefit monitoring boundary, and did not cross the futility boundary. Therefore, there was not a statistical difference in both conventional meta-analysis and TSA, but further information is required because a type II error (false negative) cannot be excluded.

The TSA reported in Figure 4b shows that the RIS (216 patients) was reached in the analysis of maximum specimen length. The cumulative Z-curve was beyond the futility boundaries did crossed both the conventional test and the benefit monitoring boundaries, thus supporting the superiority of PC-LB over EUS-LB for this specific outcome.

As reported in Supplementary Figure S5, the TSA concerning the number of CPTs showed that the RIS (2640 participants) was not reached and the cumulative Z-curve remained within conventional boundaries (*p* > 0.05) and far from both the futility and monitoring boundaries. Thus, the result from the conventional meta-analysis means that there is no effect or a lack of power.

## 4. Discussion

Although non-invasive methods showed interesting results for diagnosis and monitoring of fibrosis in chronic liver disease [2], liver biopsy is still of paramount importance in several conditions when proper histology and immunohistochemistry are required. However, traditional PC-LB techniques are limited by the risk of potentially serious complications, inter-observer variations, and sampling errors leading to false negative diagnosis [26,27]. TJ-LB might have a role in high-risk patients, such as those with coagulopathy, in antithrombotic therapy, or high-volume ascites; however, the complexity of the procedure and the potential risks for complications limit its applicability. The EUS-LB technique allows for obtaining samples from both hepatic lobes, thus improving the ability to access focal liver lesions, and EUS guidance can confirm the presence or absence of bowel, blood vessels, and biliary structures along the needle track in real time, for both lobes, greatly enhancing its safety profile. EUS-LB also minimizes the impact of ascites and body habitus on the ability to visualize and obtain liver tissue [28]. Although preliminary studies and previous meta-analyses have not shown any significant difference between EUS-LB and PC-LB, two recent RCTs [13,14] found a clear superiority of EUS-LB over PC-LB in terms of sample adequacy, in discordance with a preliminary American trial [11]. According to the GRADE methodology, only a meta-analysis of RCTs can provide high certainty in the estimates [25]. Previous systematic reviews included predominantly retrospective studies; hence, we decided to perform a meta-analysis of RCTs to try to draw definitive assumptions concerning the comparison between EUS-LB and PC-LB.

Therefore, through a meta-analysis of four RCTs, we made several key observations. First, there was no difference between the two techniques in terms of sample adequacy. Of note, the definition of sample adequacy was slightly heterogeneous across the RCTs and very restrictive, including only high-quality samples (at least 20 mm of length and 11 CPTs), hence the relatively low pooled adequacy rate (60% with EUS-LB and 51% with PC-LB). As a consequence of the different definition used in the RCTs, high heterogeneity was observed in this analysis (I^2^ = 88%); this heterogeneity consistently dropped down in the sensitivity analysis performed based on the definition of the outcome. Interestingly, the two most recent RCTs [13,14] that used the most restrictive definition as per current guidelines [1] showed a clear superiority of EUS-LB over PC-LB, although this finding should be interpreted with caution due to the very limited number of studies. The TSA confirmed the limited evidence supporting the comparison between the two techniques for this outcome, as only around 25% of the RIS was actually accrued in the RCTs conducted so far; therefore, we cannot draw definitive conclusions on the superiority of one technique over the other as further RCTs are still needed and the current finding of equal effectiveness of EUS-LB and PC-LB could be due to a type II error (false negative) because of the lack of power in the meta-analysis. As a consequence, only a conditional recommendation based on very low-quality evidence can be currently provided.

All the studies used the same needle size (19 G), which was found to outperform other sizes when performing EUS-LB in a recent RCT [29]; on the other hand, results of the subgroup analysis based on needle design (FNA vs. FNB) should be interpreted with caution as only one RCT [14] used FNA, whereas the other studies used the newer end-cutting FNB needles. Although a recent RCT found 19-G FNB to be superior to 19-G FNA [10], the RCT by Samanta et al. [14] showed very favorable results with the FNA needle, clearly superior to PC-LB; further head-to-head trials comparing different needle designs are needed in this field. Likewise, the limited number of RCTs did not enable us to perform further sensitivity analyses based on specific technical strategy for tissue sampling, such as number of actuations during needle passes or use of wet-suction biopsy. Preliminary studies [30,31] gave some indications in this regard but evidence is still too scarce to reach an agreement on the best tissue sampling technique for EUS-LB, as we have for pancreatic masses [7]. On the other hand, no different results were observed based on the different needle size used during PC-LB, whether 16 G or 18 G.

As observed in previous meta-analyses [15,16], no difference in terms of diagnostic accuracy or total specimen length was observed. Diagnostic accuracy is high with both techniques, thus confirming the high effectiveness of liver biopsy. The total specimen length and number of CPTs were also similar between EUS-LB and PC-LB. Furthermore, in the analysis of the number of CPTs, the TSA showed that the RIS was not reached and the cumulative Z-curve remained within conventional boundaries, thus meaning that the meta-analysis might still lack power. As observed in a previous meta-analysis [16], the mean maximum specimen length was significantly longer in the PC-LB group (*p* = 0.01), although with evidence of high heterogeneity. The TSA showed that the RIS was reached in this analysis and the cumulative Z-curve crossed both the conventional test and the benefit monitoring boundaries, thus supporting the superiority of PC-LB over EUS-LB in terms of maximum specimen length. Moderate quality of evidence informed this analysis due to the high heterogeneity; of note, high heterogeneity frequently characterizes the meta-analysis of continuous variables and in this case was not due to a different direction of the results of the RCTs. Therefore, we can give a definitive conclusion on the superiority of PC-LB over EUS-LB in providing longer non-fragmented specimens, although this does not lead to a superior diagnostic accuracy, as previously mentioned. Finally, no major AEs were observed in the included studies and only mild cases of abdominal pain were registered in a small group of patients after both procedures.

There are certain limitations to our study which merit further discussion. First, the number of included studies and recruited patients was low and, as aforementioned, the RIS was not reached in the primary outcome. Second, the included RCTs were unblinded, hence prone to performance bias. However, it should be noted that this bias is not avoidable in endoscopy studies as the operator cannot be blinded to the device used. Moreover, no deviations from the intended protocol were observed, hence the RCTs were considered to be at low risk of bias. Third, as already commented, some technical features such as needle design or sampling techniques could not be compared due to the lack of data. Finally, our results should be considered applicable only to parenchymal liver disease, as data on focal lesions are lacking. The preliminary results from an Italian multicenter study [12] seem to confirm the aforementioned findings even in this setting, but RCTs are needed in this field. Specific sub-analyses based on the location of tissue sampling with both methods were not feasible due to the lack of data; therefore, further studies aiming to define the safety of these techniques based on the location of sampling are needed.

## 5. Conclusions

In conclusion, EUS-LB and PC-LB appear comparable in terms of the diagnostic performance and quality of tissue, although longer non-fragmented specimens seem to be achieved more frequently with PC-LB. Further multicenter RCTs, with larger sample sizes, are warranted in order to inform the comparison between these two approaches, specifically in light of newer end-cutting EUS-FNB needles.

## Figures and Tables

**Table 1:** Baseline characteristics of included randomized controlled trials.

Study	Country	Study Period	Sample Size	Age	Gender Male	Indication (Abnormal Liver Function Tests)	Number of Needle Passes	Needle Used	Definition of Adequate Samples
Bang 2021 [11]	USA	2019–2020	EUS-LB: 21 PC-LB: 19	55 ± 15.9 53.7 ± 14.6	8 (38.1%) 6 (31.6%)	NR	2 1	19 G Acquire^®^ 16 G Biopince^®^	Specimen length ≥ 25 mm and number of CPTs ≥ 11
Ali 2023 [17]	USA	2020–2021	EUS-LB: 40 PC-LB: 40	52.5 (19–76) 53 (25–75)	13 (32.5%) 13 (32.5%)	25 (62.5%) 27 (67.5%)	2 (1–3) 3 (1–7)	19 G Acquire^®^ or Sharcore^®^ 18 G needle	Number of CPTs ≥ 11
Larino-Noia 2023 [13]	Spain	2022	EUS-LB: 44 PC-LB: 46	60.8 ± 8 58.8 ± 11.2	22 (50%) 21 (45.7%)	NR	1 (1–2) 1 (1–2)	19 G Acquire^®^ 16 G Biopince^®^	Specimen length ≥ 20 mm and number of CPTs ≥ 11
Samanta 2023 [14] ^a^	India	2020–2021	EUS-LB: 24 PC-LB: 24	37.04 ± 11.6 37.46 ± 13.9	18 (75%) 12 (50%)	9 (37.5%) 6 (25%)	NR	19 G FNA 18 G needle	Specimen length ≥ 20 mm and number of CPTs ≥ 11

Data are reported as absolute numbers (percentages) or mean (±standard deviation or with interquartile range). ^a^ Study published as conference abstract. Abbreviations: CPTs, complete portal tracts; EUS-LB, endoscopic ultrasound liver biopsy; FNA, fine-needle aspiration; NR, not reported; PC-LB, percutaneous liver biopsy.

**Table 2:** Meta-analysis of study outcomes.

Outcome	Interventions	No. of Studies	No. of Patients	Risk Ratio (95% CI)	Within-Group Heterogeneity (I^2^)
**Optimal core procurement rate**	**EUS-LB** **PC-LB**	4	129 129	1.18 (0.58–2.38) *p* = 0.65	88%
**Diagnostic accuracy**	**EUS-LB** **PC-LB**	4	129 129	1.0 (0.95–1.05) *p* = 0.88	0%
**Outcome**	**Interventions**	**No. of Studies**	**No. of Patients**	**Mean Difference (95% CI)**	**Within-group heterogeneity (I^2^)**
**Number of CPTs**	**EUS-LB** **PC-LB**	3	108 110	2.29 (−4.08 to 8.66) *p* = 0.48	90%
**Max specimen length**	**EUS-LB** **PC-LB**	4	129 129	−3.11 (−5.51 to −0.71) *p* = 0.01	75%
**Total specimen length**	**EUS-LB** **PC-LB**	3	108 110	−0.51 (−20.92 to 19.90) *p* = 0.96	95%

Abbreviations: CI, confidence interval; EUS-LB, endoscopic ultrasound liver biopsy; PC-LB, percutaneous liver biopsy.

## Data Availability

The data presented in this study are available on request from the corresponding author. The data are not publicly available because no new data were created or analyzed in this study.

## Supplementary Materials

The following supporting information can be downloaded at: https://www.mdpi.com/article/10.3390/diagnostics14121238/s1, Table S1. Risk of bias assessment and quality of included studies; Table S2. Sensitivity analysis concerning the primary outcome (sample adequacy); Table S3. Certainty assessment; Figure S1. Funnel plots for assessing the risk of publication bias concerning (a) sample adequacy; (b) diagnostic accuracy; (c) number of complete portal tracts; (d) max specimen length; (e) total specimen length; Figure S2. Forest plot comparing number of complete portal tracts between the two techniques; Figure S3. Forest plot comparing max specimen length between the two techniques; Figure S4. Forest plot comparing total specimen length between the two techniques; Figure S5. Trial sequential analysis concerning the mean number of complete portal tracts.
