# Supplementary material for: Diagnostic Yield of Endoscopic Ultrasound-Guided Liver Biopsy in Comparison to Percutaneous Liver Biopsy: A Meta-Analysis of Randomized Controlled Trials and Trial Sequential Analysis
Source: Diagnostics (Basel). 2024 Jun 12;14(12):1238. doi: 10.3390/diagnostics14121238 (PMC11203010; doi:10.3390/diagnostics14121238)
Supplement: Supplementary file 1 [file diagnostics-14-01238-s001.zip › diagnostics-3051578-supplementary.pdf]

ONLINE SUPPLEMENT

Supplementary Table S1. Risk of bias assessment and quality of included studies.

	Randomization process	Deviations from intended interventions	Missing outcome data	Measurement of the outcome	Selection of the reported result
Bang 2021	L	L	L	L	L
Ali 2023	L	L	L	L	L
Larino-Noia 2023	L	L	L	L	L
Samanta 2023	L	L	L	L	L

L, low; H, high;

Supplementary Table S2. Sensitivity analysis concerning the primary outcome (sample adequacy).

Variable	Intervention	No. of Studies	No. of patients	Risk ratio (95% CI)	Within-group heterogeneity (I^2)
**Needle used for EUS-LB**	**FNB**	3	105 vs. 105	0.93 (0.41-2.11)	88%
	**FNA**	1	24 vs. 24	2.33 (1.36-4)	--
**Needle for PC-LB**	**18G**	2	65 vs. 65	0.97 (0.20-4.79)	91%
	**16G**	2	64 vs. 64	1.34 (0.46-3.90)	92%
**Definition of adequacy**	**As per current guideline**	2	68 vs. 70	2.19 (1.54-3.11)	0%
	**Different definition**	2	61 vs. 59	0.64 (0.34-1.22)	36%

Abbreviations: CI, confidence interval; EUS-LB, endoscopic ultrasound liver biopsy; PC-LB, percutaneous liver biopsy.

Supplementary Table S3. Certainty assessment.

No. of studies	Study design	Risk of bias	Inconsistency	Indirectness	Imprecision	Publication bias	Certainty
**Adequacy**							
4	RCT	low[a]	high	high	high[b]	low	⊕◯◯◯ Very Low
**Accuracy**							
4	RCT	low[a]	low	low	high[b]	low	⊕⊕⊕◯ Moderate
**Max. specimen length**							

4	RCT	low[a]	high	low	low	low	⊕⊕⊕◯ Moderate

a. Included RCTs were unblinded but no deviations from the initial protocol were detected.
b. Wide confidence intervals crossing unity or failure to reach the optimal information size.

Supplementary Figure S1. Funnel plots for assessing the risk of publication bias concerning (a) sample adequacy; (b) diagnostic accuracy; (c) number of complete portal tracts; (d) max. specimen length; (e) total specimen length.

(a)

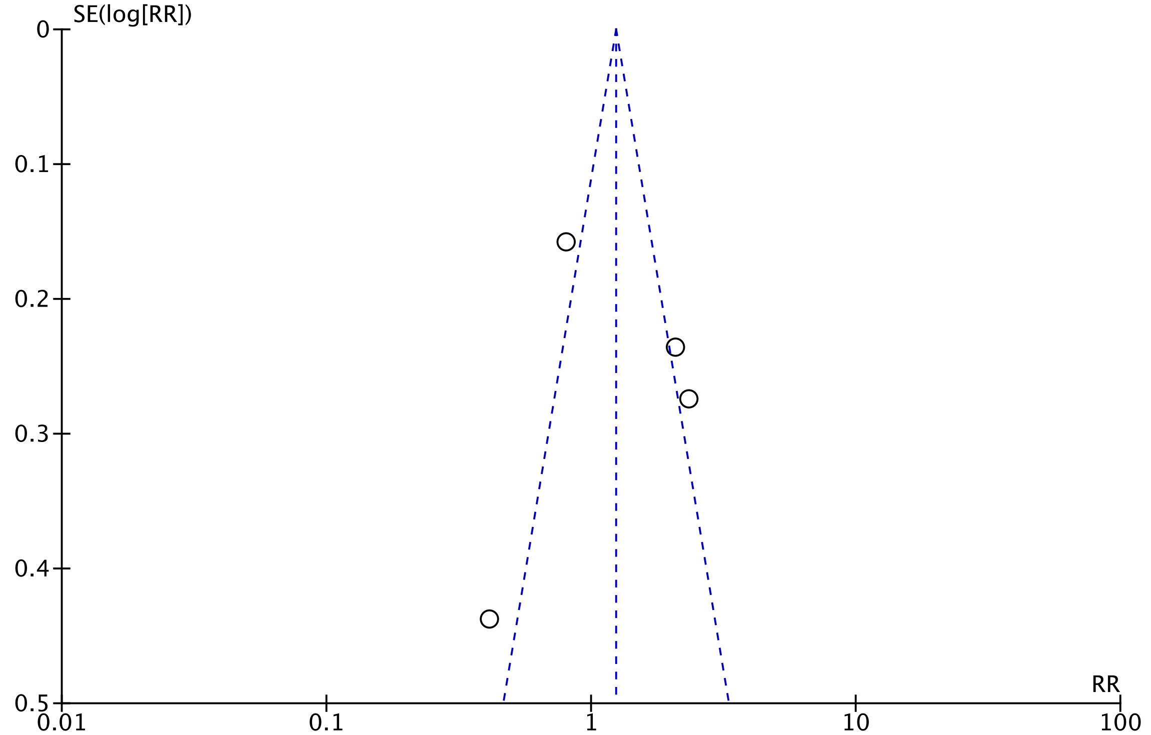

SE(log[RR])
0
0.1
0.2
0.3
0.4
0.5
RR
0.01
0.1
1
10
100

(b)

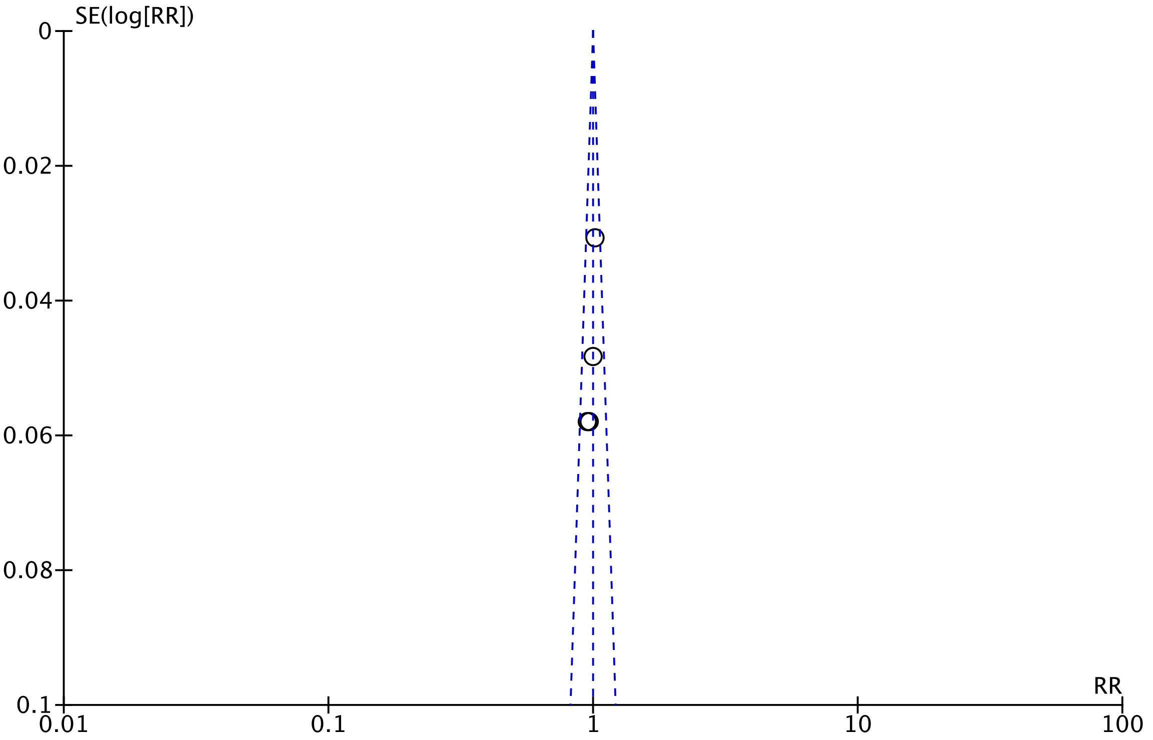

SE(log[RR])
0
0.02
0.04
0.06
0.08
0.1
RR
0.01
0.1
1
10
100

(c)

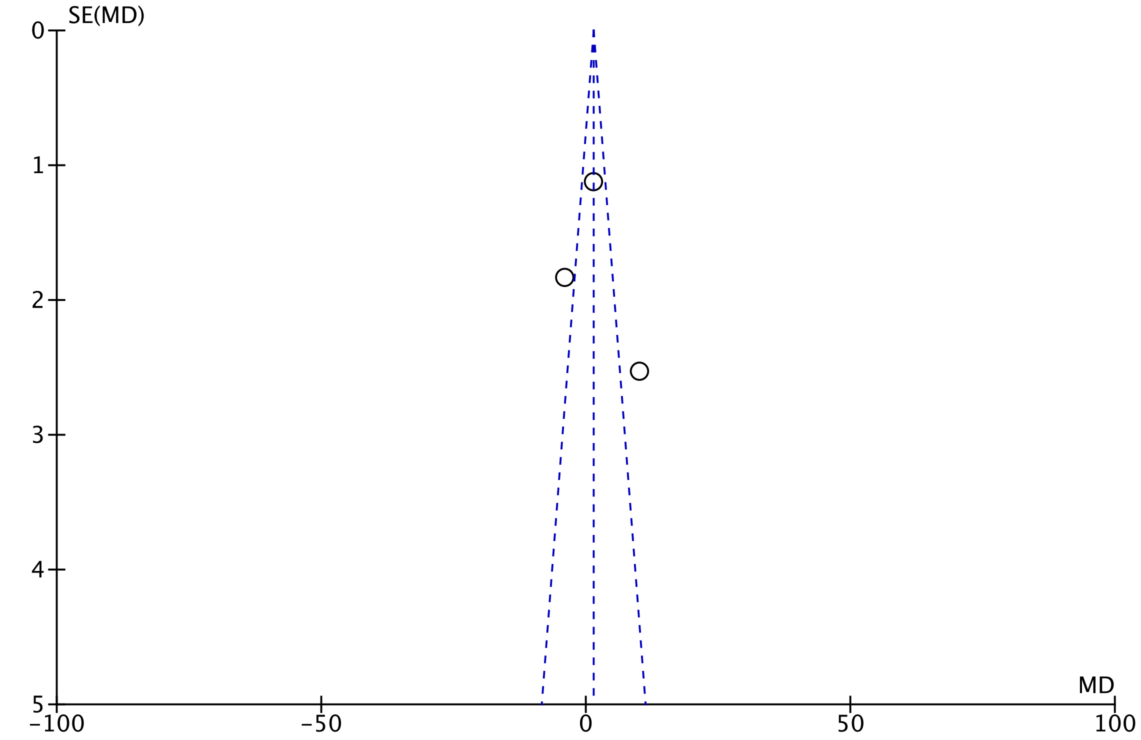

(d)

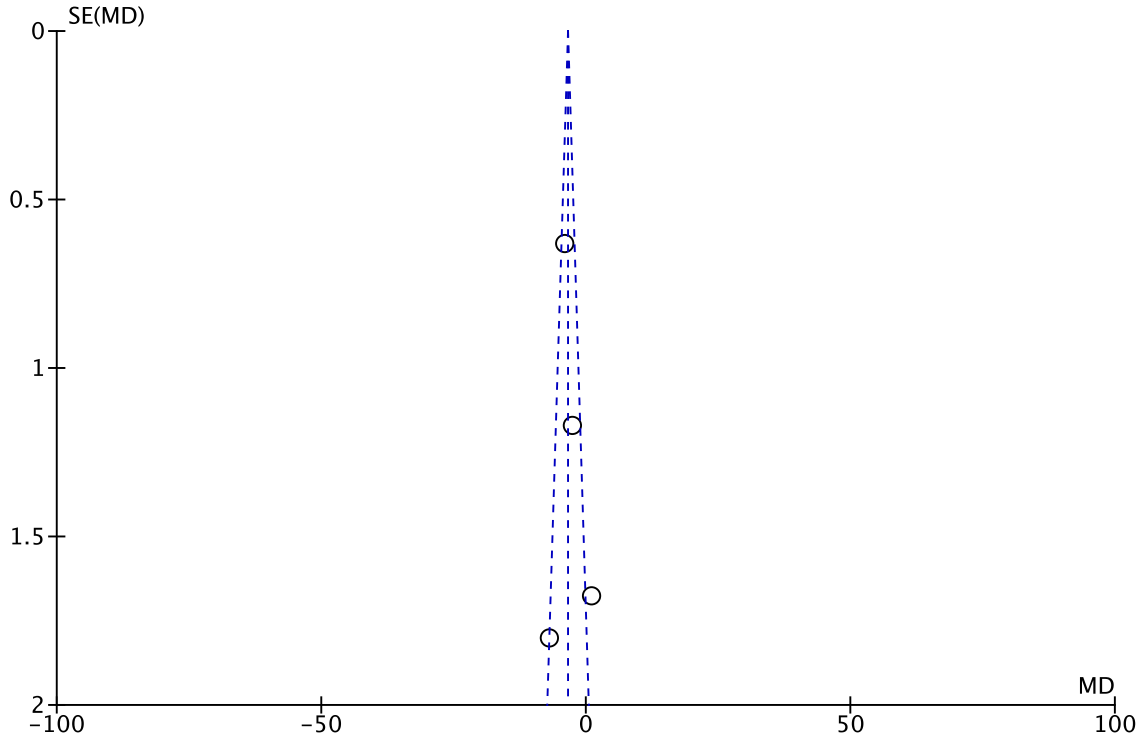

(e)

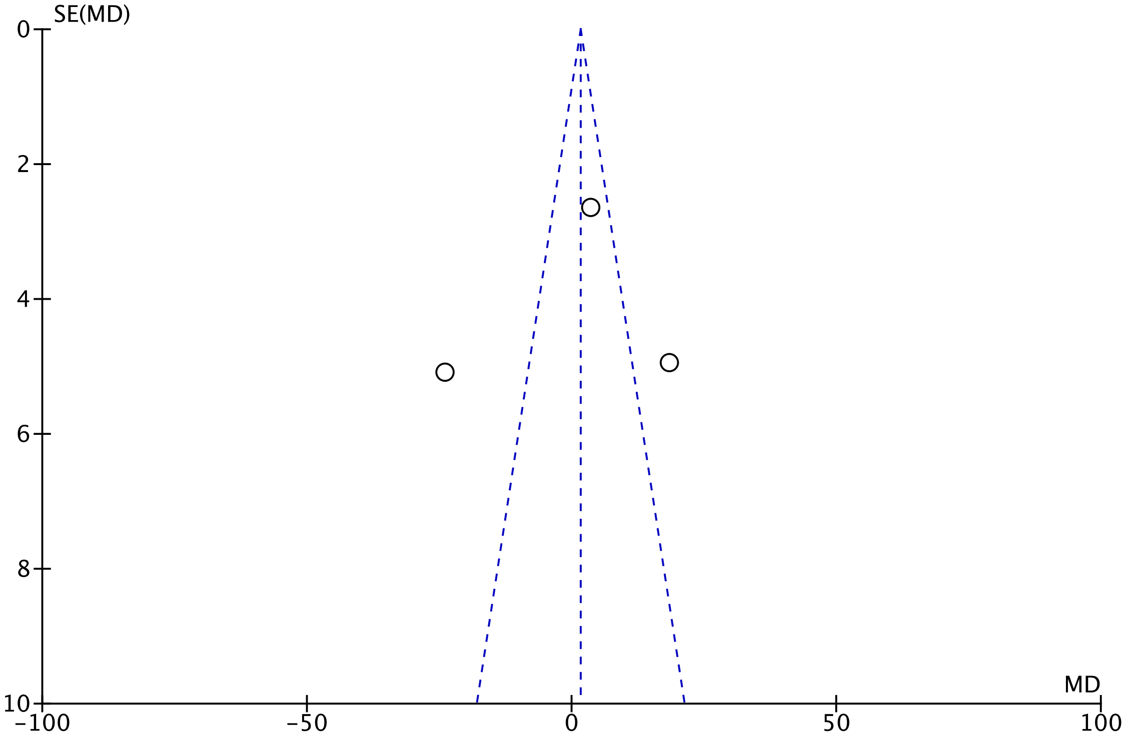

Supplementary Figure S2. Forest plot comparing number of complete portal tracts between the two techniques.

Study or Subgroup	EUS−LB Mean	EUS−LB SD	EUS−LB Total	PC−LB Mean	PC−LB SD	PC−LB Total	Weight	Mean Difference IV, Random, 95% CI
Ali 2023	13	7.5	40	17	8.8	40	33.5%	−4.00 [−7.58, −0.42]
Larino−Noia 2023	12.27	6.5	44	10.68	3.84	46	35.9%	1.59 [−0.63, 3.81]
Samanta 2023	17.5	10.8	24	7.5	6	24	30.6%	10.00 [5.06, 14.94]
**Total (95% CI)**			**108**			**110**	**100.0%**	**2.29 [−4.08, 8.66]**

Heterogeneity: $Tau^2 = 28.17$; $Chi^2 = 20.37$, df = 2 (P < 0.0001); $I^2 = 90\%$
Test for overall effect: Z = 0.70 (P = 0.48)

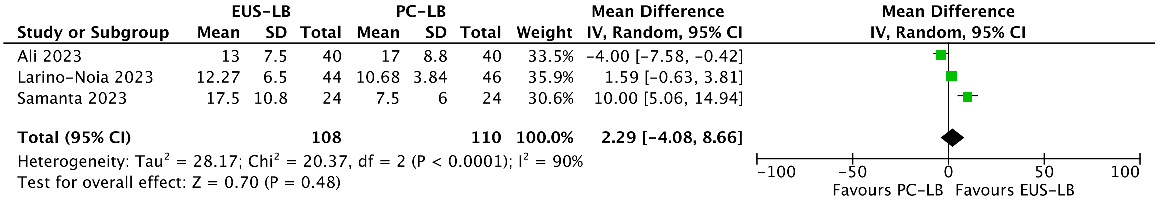

Supplementary Figure S3. Forest plot comparing max. specimen length between the two techniques.

	EUS-LB			PC-LB				Mean Difference
Study or Subgroup	Mean	SD	Total	Mean	SD	Total	Weight	IV, Random, 95% CI
Ali 2023	21	9.3	40	20	5.1	40	21.2%	1.00 [−2.29, 4.29]
Bang 2021	19.2	6.9	21	25.9	4.3	19	20.0%	−6.70 [−10.23, −3.17]
Larino-Noia 2023	8.93	3.7	44	13.02	2.03	46	32.2%	−4.09 [−5.33, −2.85]
Samanta 2023	8	2.8	24	10.5	5	24	26.6%	−2.50 [−4.79, −0.21]
**Total (95% CI)**			**129**			**129**	**100.0%**	**−3.11 [−5.51, −0.71]**

Heterogeneity: $Tau^2 = 4.27$; $Chi^2 = 11.96$, df = 3 (P = 0.008); $I^2 = 75\%$
Test for overall effect: Z = 2.54 (P = 0.01)

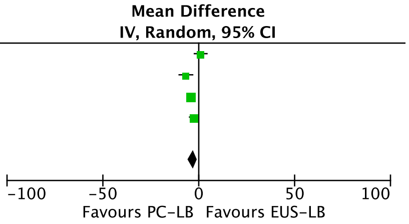

Supplementary Figure S4. Forest plot comparing total specimen length between the two techniques.

	EUS-LB			PC-LB				Mean Difference
Study or Subgroup	Mean	SD	Total	Mean	SD	Total	Weight	IV, Random, 95% CI
Ali 2023	36	28.2	40	60	15.5	40	32.6%	−24.00 [−33.97, −14.03]
Larino-Noia 2023	25.8	13.63	44	22.16	11.4	46	34.6%	3.64 [−1.56, 8.84]
Samanta 2023	33.5	23.8	24	15	4.8	24	32.8%	18.50 [8.79, 28.21]
**Total (95% CI)**			**108**			**110**	**100.0%**	**−0.51 [−20.92, 19.90]**

Heterogeneity: $Tau^2 = 306.35$; $Chi^2 = 37.53$, df = 2 (P < 0.00001); $I^2 = 95\%$
Test for overall effect: Z = 0.05 (P = 0.96)

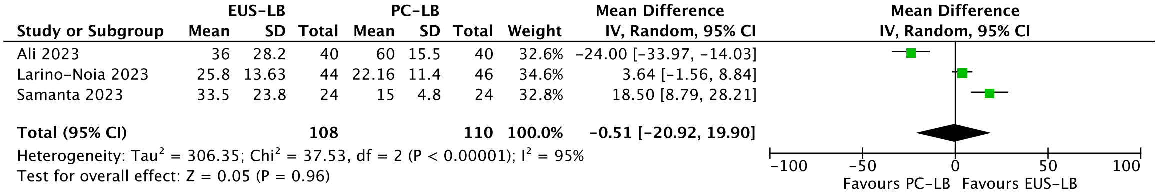

Supplementary Figure S5. Trial sequential analysis concerning the mean number of complete portal tracts.

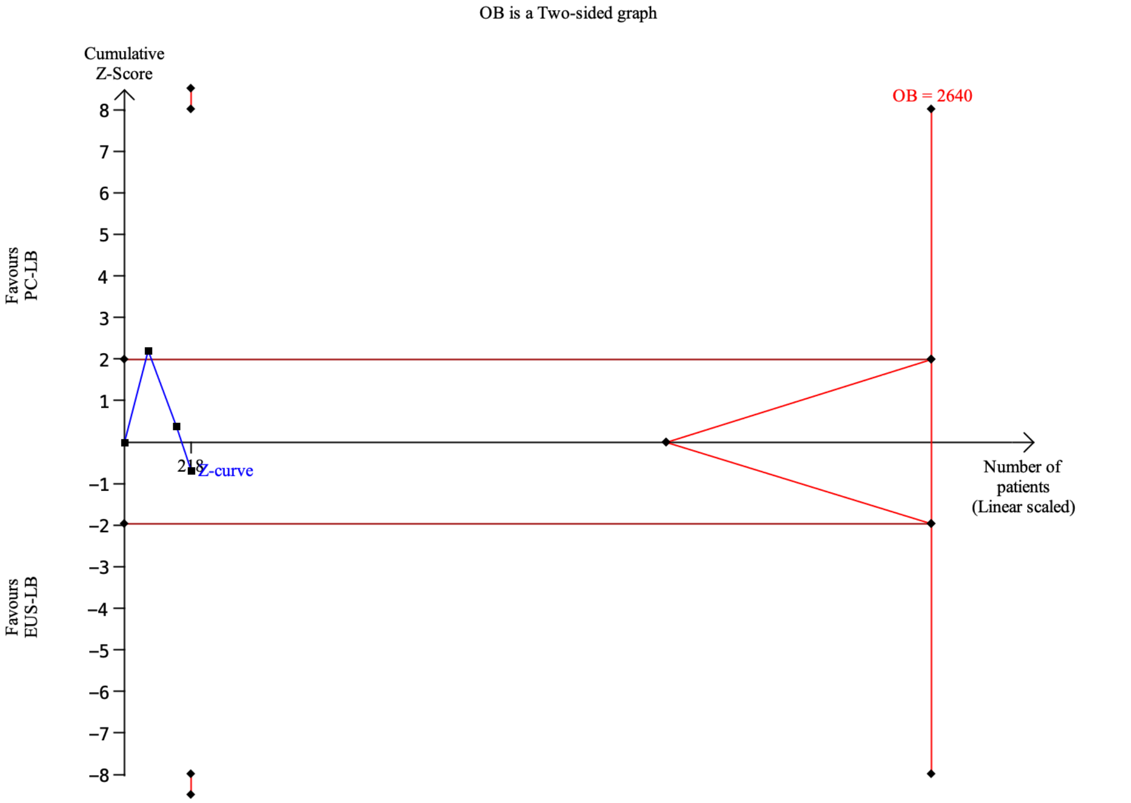

OB is a Two-sided graph
Cumulative Z-Score
8
7
6
5
4
3
2
1
−1
−2
−3
−4
−5
−6
−7
−8
Favours PC-LB
Favours EUS-LB
OB = 2640
218
Z-curve
Number of patients (Linear scaled)
